# Gold Nanorod Substrate for Rat Fetal Neural Stem Cell Differentiation into Oligodendrocytes

**DOI:** 10.3390/nano12060929

**Published:** 2022-03-11

**Authors:** Krishna Deo Sharma, Karrer M. Alghazali, Rabab N. Hamzah, Sahitya Chetan Pandanaboina, Zeid A. Nima Alsudani, Malek Muhi, Fumiya Watanabe, Guo-Lei Zhou, Alexandru S. Biris, Jennifer Yanhua Xie

**Affiliations:** 1 Molecular Biosciences Graduate Program, Arkansas State University, State University, AR 72467, USA; krishna.sharma@smail.astate.edu (K.D.S.); gzhou@astate.edu (G.-L.Z.); 2 Center for Integrative Nanotechnology Sciences, University of Arkansas at Little Rock, Little Rock, AR 72204, USA; karrer@nushores.com (K.M.A.); rnhamzah@ualr.edu (R.N.H.); zanima@ualr.edu (Z.A.N.A.); mamuhi@ualr.edu (M.M.); fxwatanabe@ualr.edu (F.W.); 3 NuShores BioSciences LLC, Little Rock, AR 72211, USA; 4 Arkansas Biosciences Institute, Arkansas State University, Jonesboro, AR 72401, USA; drpandana@gmail.com; 5 Department of Biological Sciences, Arkansas State University, State University, AR 72467, USA; 6 Department of Basic Sciences, New York Institute of Technology College of Osteopathic Medicine, Arkansas State University, Jonesboro, AR 72401, USA

**Keywords:** gold nanorod, rat fetal neural stem cells, differentiation, oligodendrocytes

## Abstract

Gold nanorods (AuNRs) have been proposed to promote stem cell differentiation in vitro and in vivo. In this study, we examined a particular type of AuNR in supporting the differentiation of rat fetal neural stem cells (NSCs) into oligodendrocytes (ODCs). AuNRs were synthesized according to the seed-mediated method resulting in nanorods with an aspect ratio of around 3 (~12 nm diameter, 36 nm length) and plasmon resonance at 520 and 780 nm, as confirmed by transmission electron microscopy (TEM) and UV-vis spectroscopy, respectively. A layer-by-layer approach was used to fabricate the AuNR substrate on the functionalized glass coverslips. NSCs were propagated for 10 days using fibroblast growth factor, platelet-derived growth-factor-supplemented culture media, and differentiated on an AuNR or poly-D-lysine (PDL)-coated surface using differentiation media containing triiodothyronine for three weeks. Results showed that NSCs survived better and differentiated faster on the AuNRs compared to the PDL surface. By week 1, almost all cells had differentiated on the AuNR substrate, whereas only ~60% differentiated on the PDL surface, with similar percentages of ODCs and astrocytes. This study indicates that functionalized AuNR substrate does promote NSC differentiation and could be a viable tool for tissue engineering to support the differentiation of stem cells.

## 1. Introduction

Myelin sheaths are vital to the rapid or instantaneous functions of the nervous system, including sensory perception, motor response, and cognition. Oligodendrocytes (ODCs) are the primary glial cells in the vertebrate central nervous system (CNS) that form the myelin sheath around axons. The myelin sheath is a lipid bilayer (plasma membrane of ODCs) that surrounds the axons of neurons to help insulate signals to ensure fast conduction of the electrical impulses through the flow of ions. It also protects the axons and provides the trophic support necessary for axonal survival [1]. The importance of ODCs to CNS functionality is proven by neuropathological conditions in which the myelin sheath is impaired due to ODC degeneration, leading to demyelinating diseases, such as multiple sclerosis or myelitis.

Like CNS neurons, ODCs have limited ability to divide and regenerate new cells. Therefore, disease- or injury-induced functional loss to the CNS cannot yet be cured. Cell therapy and tissue engineering could provide a possible therapeutic option to regenerate neural tissue or replace lost neural cells in CNS disorders and injuries. In this regard, nanomaterial-based scaffolds and synthetic polymers designed to mimic the extracellular environment of the CNS have been widely studied in stem cell transplantation and neural cell regeneration experiments [2,3,4]. Gold nanomaterial surfaces have been explored for neural-based biomedical applications since they have significant advantages, including biocompatibility, optical properties, electrical conductivity, and ability to generate surface-enhanced Raman spectroscopy (SERS), photothermal (PT), and photoacoustic (PA) effects [5,6].

Nanomaterials, particularly gold nanomaterials, have proved to be extremely scientifically important building blocks towards the development of an artificial surface for enhanced surface proliferation and differentiation [6,7,8]. Previously, we reported the use of gold nanorods and their derivatives for SERS-based visualization of complex 3D systems [9,10,11], as well as creation of films for the differentiation of rat embryonic cortical neural stem cells (NSCs) into neurons on the surface of gold nanorods (AuNRs), demonstrating that AuNRs provide a conducive and optimum surface towards cellular attachment and differentiation [6]. In the present study, we evaluated the targeted differentiation of rat NSCs into ODCs over three weeks, expression of functional protein by newly differentiated ODCs, and integrin expression in differentiated cells on AuNRs.

## 2. Materials and Methods

### 2.1. Materials

DI water was used in all steps unless otherwise specified. (III) chloride trihydrate (99%), sodium borohydride (99%), L-ascorbic acid (98%), N-ethyl-N′-(3-(dimethylamino)propyl)carbodiimide (EDC), and N-hydroxysuccinimide (NHS) were bought from Sigma-Aldrich (St. Louis, MO, USA). Cetyltrimethylammonium bromide (CTAB, 99%), 3-aminopropyltriethoxysilane, and silver nitrate were purchased from Fisher Scientific (Hampton, NH, USA). HS-PEG (MW 5000), HS-PEG-COOH (MW 3000), and HS-PEG-NH_2_ (MW 3000) were bought from Nanocs Inc (New York, NY, USA). 12-mm glass coverslips were purchased from Fisher Scientific. All other general lab reagents were obtained from Sigma-Aldrich.

Rat fetal NSCs were purchased from Gibco, (Waltham, MA, USA). These cells were harvested and cryopreserved from the cortex of Sprague Dawley rats at embryonic day 14 (E14). They retained their undifferentiated characteristics upon propagation in StemPro serum-free medium and could differentiate into neurons, astrocytes, and ODCs in the absence of growth factors in the culture medium. Dulbecco’s modified Eagle medium (DMEM/F-12), neurobasal medium, L-glutamine, basic fibroblast growth factor (bFGF), StemPro neural supplement, Glutamax, B-27 supplement, and epidermal growth factor (EGF) were also purchased from Gibco, USA. 3, 3′ 5-triiodothyronine (T3) and poly-D-lysine (PDL) were purchased from SIGMA, (Waltham, MA, USA), and Matrigel was purchased from Corning, (Corning, NY, USA). Recombinant human-platelet-derived growth factor (PDGF-AA) was commercially obtained from Millipore, (Burlington, MA, USA). Primary antibodies were purchased as follows: mouse monoclonal receptor interacting protein (RIP) (from Developmental Studies Hybridoma Bank, Iowa City, IA, USA); rabbit anti-myelin basic protein (MBP) and rabbit anti-nestin (from Abcam, Waltham, MA, USA); rabbit monoclonal anti-glial fibrillary acidic protein (GFAP) and mouse anti-focal adhesion kinase (FAK) from Sigma, USA. Secondary antibodies, Alexa fluor 488 anti-mouse IgG and Alexa fluor 560–590 anti-rabbit IgG, were obtained from Thermo Fisher, USA. Nuclear stain 4′,6-diamino-2-phenylindole dihydrochloride (DAPI) was commercially obtained from Invitrogen, (Waltham, MA, USA).

### 2.2. Fabrication and Functionalization of AuNR Surfaces

AuNRs with an aspect ratio of around 3 (average diameter ~12 nm; average length ~36 nm) were synthesized according to the silver ion-assisted, seed-mediated method developed by Nikoobakht [7]. The thiol–gold binding reaction was used to modify the surface chemistry with different functional groups (HS-PEG-X, where X = NH_2_ or COOH), as reported previously [8,9]. Their stability under various conditions was studied and presented in an earlier manuscript [12].

### 2.3. Fabrication of Functionalized Substrate Using Functional Silane Compounds

Glass coverslips were used as the base for the functionalized substrate. Briefly, 12-mm glass coverslips were washed with 5% HCl for 12 h to eliminate non-binding metal ions, such as potassium, sodium, and calcium. Next, a high density of hydroxyl groups was generated over the glass surface by treatment with piranha solution (mixture of 25% sulfuric acid and 30% hydrogen peroxide) for about 2 h. Then, the treated coverslips were washed with DI water. To cover the glass substrate with NH_2_, a silylation protocol was adopted [13]. Briefly, a mixture of absolute ethanol:DI water:3-aminopropyltriethoxysilane (95:3:2 %*v*/*v*) was prepared. Then, the previously cleaned glass coverslip was immersed in this mixture with vigorous stirring for about 2 h. A mixture of water/ethanol was used to wash the coverslips to remove excess silane compounds. Finally, the functionalized coverslips were cured by incubation in a preheated oven at 110 °C for 30 min.

### 2.4. Amino-Functionalized Coverslip Coating with Functionalized AuNRs

**Figure 1:**
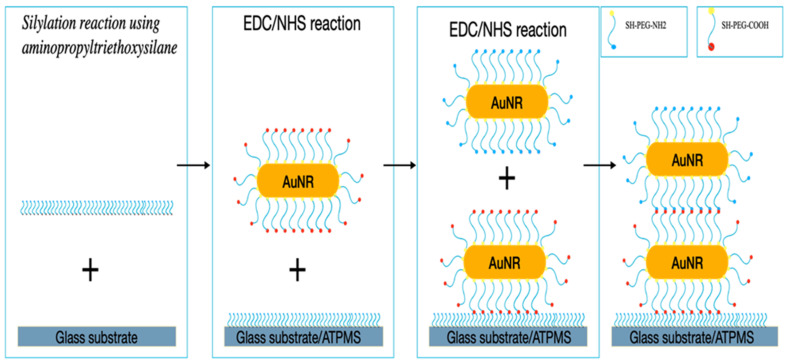
Illustration of the procedures used to produce the AuNR coatings.

A layer-by-layer approach was used to construct AuNRs on the functionalized glass coverslip, utilizing the coating protocol adopted from our previous work [8]. Briefly, as shown in Figure 1, we deposited the first layer of the AuNRs-SH-PEG-COOH over the amino-functionalized coverslip via EDC/NHS, then removed the excess chemicals and washed the substrate extensively with DI water several times to remove the unbound AuNRs-SH-PEG-COOH. After that, the second coating of AuNRs-SH-PEG-NH_2_ was deposited using the same EDC/NHS protocol, followed by the same extensive washing procedures described above.

### 2.5. Characterization of AuNRs

The AuNRs were characterized following the procedures reported by Alghazali et al., (2017) [8]. The morphology of AuNRs was analyzed by transmission electron microscopy (TEM) (JEOL USA JEM2100F, Peabody, MA, USA) with an accelerating voltage of 80 kV and by scanning electron microscopy (SEM) (JEOL JSM7000F). To conduct TEM analysis, we deposited a few drops of AuNRs and functionalized AuNRs suspended in ethanol on holey-carbon-coated copper grids. The grids were allowed to dry for 30 min at room temperature before being inserted into TEM. For SEM analysis, AuNRs were deposited on glass plates, which were in turn coated with 3 nm thick carbon films to gain electrical conductance. The UV-vis-NIR spectra of the substrates were recorded using a Shimadzu UV 3600 (Shimadzu Scientific Instruments, Columbia, MD, USA) spectrophotometer between 400 and 1000 nm, which determined the transverse and longitudinal peaks of the AuNRs, both in solution (500 µg/mL AuNRs) and on the glass substrate (AuNR thin film). Surface hydrophilicity analysis was conducted on the control plain glass coverslip and the fabricated AuNRs to find the average water contact angles (WCA) using an EasyDrop (DSA1) device (Kruss GmbH, Hamburg, Germany); on three random spots per sample, 8-μL DI water droplets were gently dispensed from a stainless-steel needle via an automated, computer-controlled syringe.

### 2.6. Rat NSC Culture

To obtain an adequate amount of neural progenitor cells (NPCs) for differentiation experiments from the same lot, we propagated the rat NSCs following the protocol by Pandanaboina et al. [6]. In brief, rat NSCs were maintained in cell culture in StemPro serum-free medium supplemented with bFGF (20 ng/mL) and EGF (20 ng/mL) in T-75 flasks coated with CTS™ CELLstart™ (1:100) substrate following instructions from the supplier to obtain NPCs for up to two passages. After the second passage, cells were frozen and stored in liquid nitrogen. All the cultures were maintained at 37 °C and 5% CO_2_ in a humidified environment.

### 2.7. Differentiation of NPCs

We followed our established protocol to differentiate NPCs into ODCs [14,15]. Passage II NPCs were seeded onto a CELLstart-coated T-25 flask (200,000 cells/flask) that consisted of a complete StemPro serum-free medium supplemented with bFGF (15 ng/mL). Twenty-four hours later, the medium was replaced with the StemPro medium, which contained bFGF (10 ng/mL) and PDGF-AA (10 ng/mL). Every third day, the medium in the flask was substituted with fresh medium for seven days. After the 7th day, the medium was then replaced with a StemPro medium that contained only bFGF (15 ng/mL) to generate late-stage ODC progenitor cells (OPCs), and the culture was maintained for another two days.

The OPCs were then cultured on PDL or AuNR surfaces. To prepare the PDL coating, 6-well glass-bottom plates were coated with 80 μg/mL PDL and incubated overnight at room temperature. The next day, the PDL solution was removed, and the dishes were rinsed with sterile nanopure water to remove excess PDL. The dishes were allowed to air dry in a sterile environment in the safety hood. To prepare the AuNR coating, AuNR substrates were individually placed in each well of 6-well glass-bottom plates and exposed to UV light for 30 min.

Next, 100 µL of cell suspension containing about 20,000 OPCs was added to each well, and the plates were placed in an incubator for the cells to attach to the AuNR or PDL-coated glass surface. After 20–30 min of incubation, 1 mL of prewarmed complete StemPro medium containing bFGF (15 ng/mL) was added to each well. On the next day, the propagation medium was replaced with the differentiation medium, which contained 1X neurobasal medium, 2% B-27 neural supplement, 2 mM glutamax supplement, and 30 ng/mL T3. Every third day, two-thirds of the medium was replaced with a freshly prepared and prewarmed medium. The cultures were maintained at 37 °C and 5% CO_2_ for 1–3 weeks.

### 2.8. Immunofluorescence Staining of Differentiated Cells

The cells were fixed using 4% paraformaldehyde and blocked with phosphate-buffered saline with Tween 20 solution that contained bovine serum albumin and glycine. Differentiated cells were then analyzed by immunocytochemical staining with primary antibodies mouse anti-RIP (1:200), rabbit anti-nestin (1:200), rabbit anti-MBP (1:200), mouse anti-FAK (1:200), and rabbit anti-GFAP (1:200), followed by goat anti-mouse Alexa Fluor 488 (green) and goat anti-rabbit Alexa Fluor 594 (red) conjugated secondary antibodies. Lastly, the cells were counterstained with DAPI to stain the nuclei (blue).

Immunolabeled cells were scanned (22 × 20 montage at 10× magnification for each well) using the Cytation5 imaging system (BioTek, Santa Clara, CA, USA), and cells were quantified in an unbiased manner using Gen5 3.05 Software (BioTek). DAPI staining was used to determine the total cell count.

### 2.9. Statistical Analysis

Data collected from three independent experiments with six wells/experiment was plotted and presented as mean ± SD. One-way analysis of variance (ANOVA) post hoc Tukey’s multiple comparison test or two-way ANOVA post hoc Dunnett’s multiple comparison test was employed using GraphPad Prism 8.0 software (GraphPad Software, San Diego, CA, USA). For our studies, *p* < 0.05 was considered statistically significant.

## 3. Results

### 3.1. AuNR Characterization

**Figure 2:**
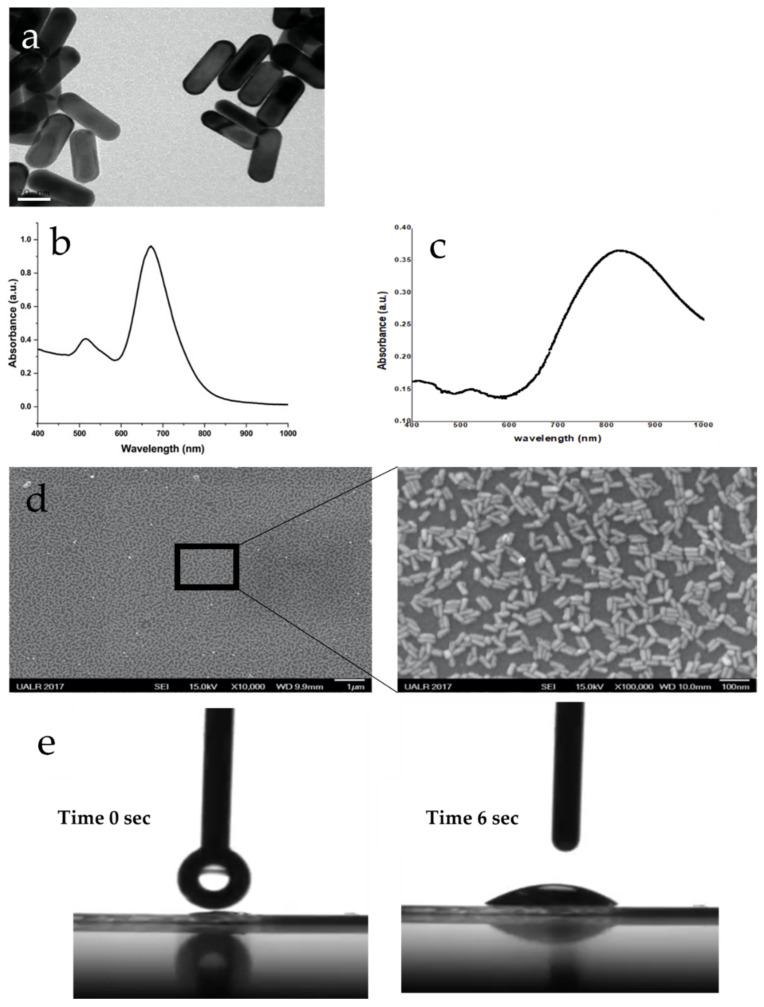
Characterization of fabricated AuNR substrate. (**a**) Representative TEM images of AuNRs. Scale bar: 20 nm; (**b**) representative UV-vis spectrum of AuNRs; (**c**) representative UV-vis spectrum of AuNR substrate; (**d**) representative SEM images of the glass coated with AuNRs. Scale bars: 1 μm left panel, 100 nm right panel; (**e**) representative contact-angle measurement for the AuNR substrate.

AuNR substrate fabrication was monitored and characterized using various characterization methods. TEM imaging (Figure 2a) confirmed the successful fabrication of AuNRs with an aspect ratio of around 3 (majority with ~12 nm average diameter, ~36 nm average length). The surface plasmon resonance (transverse and longitudinal) of the AuNRs before and after fabrication of the AuNR surface was confirmed by UV-vis spectroscopy, which showed that functionalized AuNRs in solution had surface plasmon resonance at 520 nm and 780 nm, as shown in Figure 2b. UV-vis also confirmed the presence of the AuNRs on the glass substrate, as both transverse and longitudinal surface plasmon resonance peaks were recorded on the fabricated AuNR substrate (Figure 2c). Furthermore, the coating uniformity and the presence of the AuNRs were investigated using SEM and WCA techniques. The SEM images show a relatively uniform coating of AuNRs over the glass substrates (Figure 2d). Finally, the WCA measurements demonstrate the hydrophilic characteristic of the fabricated AuNR substrate (recorded contact angle = 52.1, STDV ± 2.7) (Figure 2e), which is directly related to the chemical profile of the surface (the presence of the PEGylated coating). We previously reported that the zeta potential of pure AuNRs was −15 mV that and shifted to +24 mV after functionalization with HS-PEG-NH_2_, conforming a positive charge on the external surface due to terminal amine groups [8].

### 3.2. Differentiation of NPCs on AuNR and PDL Surfaces

Embryonic NSCs can undergo multiple self-renewal cycles and have the potential to differentiate into neurons, astrocytes, or oligodendrocytes [16]. In this study, passage II NSCs were propagated using a medium supplemented with mitogens, bFGF, and EGF for 10 days [6], and the impact of AuNR and PDL substrates on the differentiation of NPCs into ODCs was evaluated for up to three weeks. Cells were fixed and immunostained using markers for ODCs (RIP and MBP), astrocytes (GFAP), and stem cells (nestin), as well as nuclear marker DAPI (for total cell count) at weeks 1, 2, and 3.

**Figure 3:**
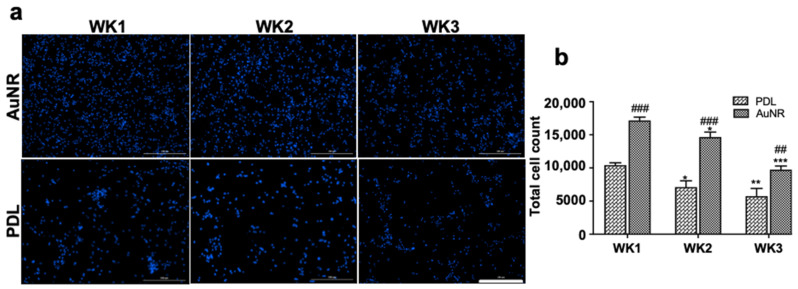
Cell count at the end of weeks 1–3. (**a**) Fluorescent images of DAPI stained cells showed that the NPCs were attached well on the AuNR and PDL surfaces. Although fewer cells survived and were attached to the substrate at week 2 and 3 than at week 1, the number of attached cells were always higher on the AuNR surface than the PDL surface (**b**). Scale bar: 200 μm. Two-way ANOVA was performed, followed by Dunnett’s multiple comparison test. * *p* < 0.1, ** *p* < 0.01, *** *p* < 0.001 compared to the same surface at week 1; ## *p* < 0.01, ### *p* < 0.001 compared to PDL.

Similar to our previous report [6], the results of this study also showed that the AuNR surface was not toxic to the cells, and cells were attached well. About 85% of cells were attached on the AuNR surface, which was significantly higher than on the PDL-coated surface (50%) at week 1 (Figure 3). Compared to week 1, only 50% of total cells remained attached at week 3, probably because cells do not stay healthy and die in the absence of growth factors in the culture medium in long-term cell cultures. However, the rate of cell death, as determined by the total number of DAPI+ cells at the end of each time point, was comparable between AuNR and PDL surfaces.

**Figure 4:**
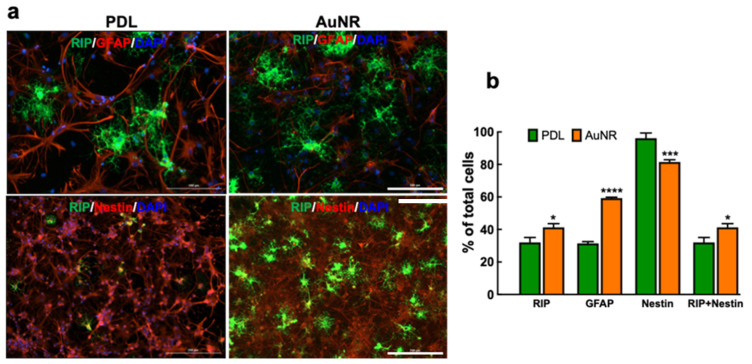
In vitro differentiation of NPCs into ODCs on PDL and AuNR substrate at week 1. (**a**) Immunostaining of NPCs with markers for oligodendrocyte (RIP, green), astrocyte (GFAP, red), intermediate filament protein (nestin, red), and nuclear stain (DAPI, blue). Scale bars: 100 µm (top), 200 µm (bottom). (**b**) Quantitative comparison of the percentage of cells expressing RIP, GFAP, and nestin on PDL and AuNR. Data show mean ± SD; comparison by one-way ANOVA with Tukey post hoc analysis; * *p* < 0.05, *** *p* < 0.001, **** *p* < 0.0001 compared to the same cell type on PDL surface.

At week 1, almost all cells differentiated into either ODCs or astrocytes on AuNR substrate; in comparison, 64% of total cells on the PDL surface were differentiated into these two glial lineages (Figure 4). About 41% and 32% of the whole cells on AuNR and PDL surfaces were RIP+ ODCs, and about 59% and 31% of the total cells were GFAP+ astrocytes on AuNR and PDL surfaces, respectively. All cells were nestin+ on PDL, showing stemness of the cells, whereas 82% of cells expressed nestin on AuNR. We did not use any marker for neurons because this culture condition favors predominant ODC differentiation, as shown in our previous report [14].

**Figure 5:**
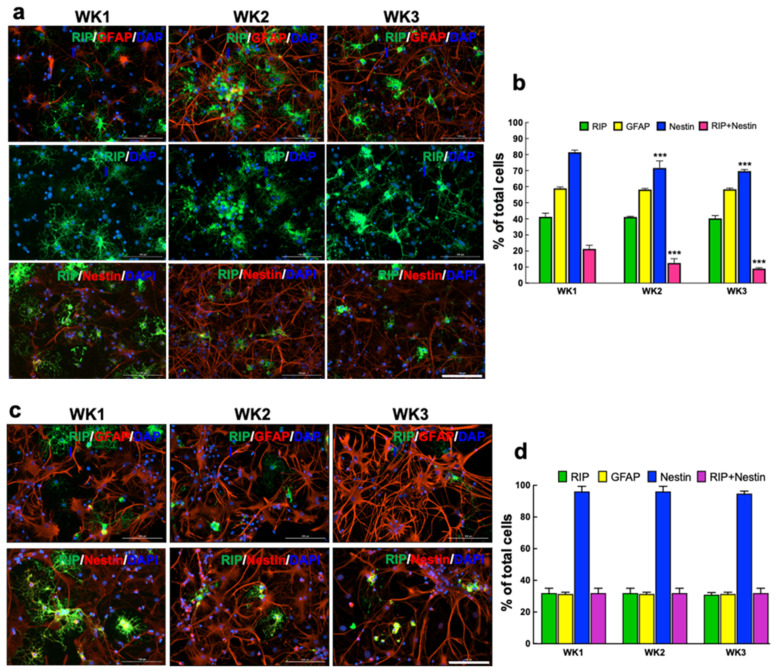
In vitro differentiation of NPCs into ODCs on AuNR substrates and PDL across three weeks. Immunostaining of NPCs differentiated on AuNR (**a**) and PDL (**c**) substrates at week 1, 2, and 3, using markers for oligodendrocyte (RIP, green), astrocyte (GFAP, red), intermediate filament protein (nestin, red), and nuclear stain (DAPI, blue). Scale bars: 100 µm. Quantitative comparison of the percentage of cells expressing RIP, GFAP, and nestin at different time points on AuNR (**b**) and PDL (**d**) surfaces. Data show mean ± SD; comparison by two-way ANOVA with Dunnett analysis; *** *p* < 0.001 compared to the same cell type at week 1.

Furthermore, we performed a longitudinal study to compare the chronological changes of NPC differentiation on PDL and AuNR substrates across three weeks of culture. The most significant difference was the number of nestin+ cells on AuNR substrates, which gradually decreased across three weeks of culture (Figure 5a,b). The number of ODCs expressing RIP and nestin simultaneously decreased significantly in weeks 2 (12%) and 2 (9%) compared to week 1 (21%). There was no change in the number of RIP+ or GFAP+ cells between the three time points. No significant changes in the number of RIP+, GFAP+ or nestin+ cells were observed across three weeks of cultures on PDL (Figure 5c,d).

### 3.3. Evaluation of Myelin Basic Protein (MBP) and Integrin Expressions

**Figure 6:**
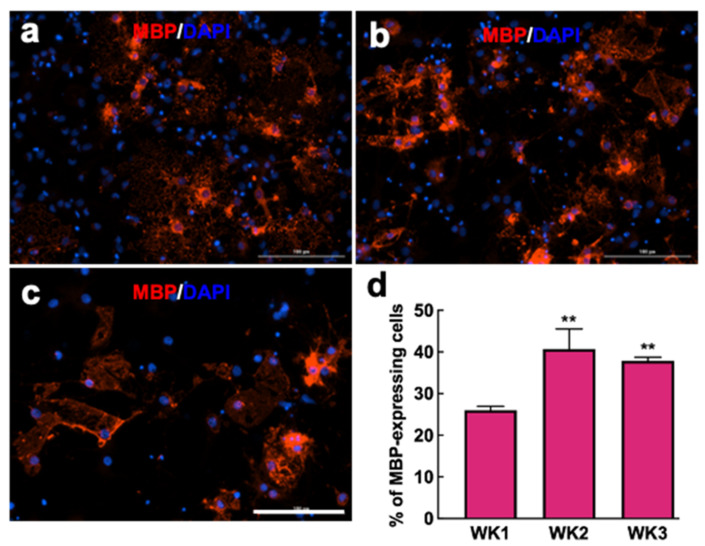
Evaluation of MBP expression by newly differentiated ODCs. Fluorescence images of NPCs differentiated on AuNR substrate at (**a**) week 1, (**b**) week 2, and (**c**) week 3 culture, stained for mature oligodendrocyte marker MBP. Scale bars: 100 µm. (**d**) Quantitative comparison of the percentage of cells expressing MBP at various time points. Data show as mean ± SD; comparison by one-way ANOVA with Tukey post hoc analysis; ** *p* < 0.001 compared to week 1.

MBP is a functional protein of myelinating ODCs that is present on mature ODCs. We evaluated the expression of MBP in newly differentiated ODCs on AuNR at different time points. As shown in Figure 6, the number of MBP+ cells was significantly higher in week 2 and 3 compared to week 1. Slightly more than half of ODCs expressed MBP at week 1, whereas all newly differentiated ODCs expressed MBP at week 2. This finding correlates well with the reduction in Rip+/nestin+ co-labeling for weeks 2 and 3, suggesting the maturation and gaining functionality of the newly differentiated ODCs.

**Figure 7:**
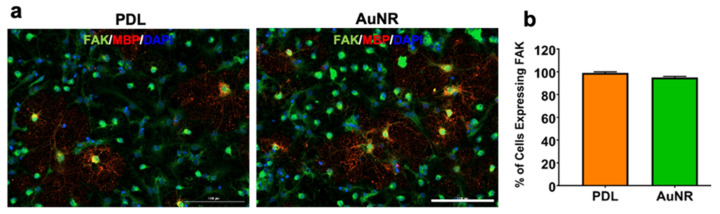
Determination of integrin activation in differentiated NPCs. (**a**) Fluorescence images of NSCs differentiated on PDL and AuNR substrates for one week and stained for FAK (green), MBP (red), and DAPI (blue). Scale bars: 100 µm. (**b**) Quantitative comparison of the percentage of cells expressing FAK and MBP.

Integrin expression has been postulated to play pivotal roles in ODC differentiation [17]. We next sought to determine integrin activation via FAK expression in differentiating NPCs on AuNR substrates and PDL-coated culture surfaces because integrin receptors are essential for in vitro growth and differentiation of ODCs [17]. NPCs were differentiated using an ODC-promoting medium on AuNR and PDL for one week, and the immunostaining results showed that almost 100% of the cells on AuNR and PDL expressed FAK (Figure 7).

## 4. Discussion

NSC transplantation is considered a potential treatment option for neurodegenerative disorders and CNS injuries, owing to the fact that NSCs can multiply and differentiate into neural cells, such as neurons, astrocytes, and oligodendrocytes [16]. Several preclinical studies have found that transplanted NSCs protect host neurons and promote functional recovery in CNS injuries [18,19]. Among the most significant challenges in using stem cell therapy for medical applications are the inhospitable environments at the lesion/implantation sites that hinder grafted cell survival, successful differentiation of the grafted cells into the preferred phenotypes, as well as the formation of working and bioactive connections between the host and grafted cells. Applications of nanomaterials in tissue engineering have recently received significant attention in an effort to overcome these challenges associated with stem cell therapy. Among several nanostructure types, gold nanoparticles have been studied for various types of tissues, including neural, bone, cartilage, cardiac, and skin tissue [2]. Furthermore, the biocompatibility, excellent tunability, and plasmonic properties of AuNRs have enabled their wide use in biomedical applications [20].

AuNRs with an aspect ratio of about three were synthesized by seed-mediated methods, and their surface chemistry was modified with NH_2_ or COOH functional groups. AuNRs were constructed on the functionalized glass coverslips using a layer-by-layer approach. Embryonic rat NSCs were cultured on the AuNR surface and differentiated into ODCs with appropriate growth factors. NSCs were primed for ODC differentiation following our previous protocol [14]. Those cells were then cultured on the AuNR fabricated surface and maintained in ODC differentiation medium for three weeks. Cells plated on the AuNR surface remained attached, and the integrity of the AuNR surface remained the same throughout the culture period. These observations are consistent with the results of our previous study, wherein we reported neuronal differentiation of NSCs on the same AuNR surface [6]. We attribute this result to the positively charged AuNR surface and the firm attachment of AuNRs to the glass coverslips.

The size of nanoparticles and their surface charges due to surface modification are essential properties of nanoparticles that significantly influence cytotoxicity. Asati et al., showed that neutral or positively charged cerium oxide nanoparticles (3–4 nm) entered different cells and could be toxic to the cells depending on the localization of the nanoparticles in the cytoplasm [21]. In the present study, the first layer of AuNRs-SH-PEG-COOH and the second layer of AuNRs-SH-PEG-NH_2_ were coated over the amino-functionalized coverslips so that the AuNRs remained attached to the coverslips, preventing internalization of the AuNR particles into the cells and thus preventing AuNR cytotoxicity.

To our knowledge, this is the first study reporting ODC differentiation of NSCs on an AuNR surface. In 2019, we have used the same AuNR substrate to differentiate NSCs and mesenchymal stem cells into neurons and astrocytes [6,8]. Collectively, these studies support our findings that an AuNR substrate provides a conducive surface for cell attachment and differentiation. Among the total population of differentiated and undifferentiated cells, about 41% were RIP+ ODCs on the AuNRs surface.

Previously, we reported more than 90% of NSCs differentiated into ODCs on Matrigel-coated glass coverslips [14]. However, only 41% of cells differentiated into ODCs on AuNRs using the same culture protocol. The underlying reason for the reduced differentiation of NSCs into ODCs on AuNR substrate needs to be explored. It is possible that ODC differentiation may be enhanced by coupling AuNRs with growth factors/biomolecules. Gold nanoparticles (AuNPs) conjugated with retinoic acid have been shown to improve neural differentiation of human adipose-derived stem cells [22]. Huang et al., showed the catalytic effect of AuNPs on nicotinamide adenine dinucleotide (NADH) in a cell-free system. They found that the fluorescence of NADH was quenched when AuNPs were mixed with NADH, possibly due to oxidation of NADH to NAD+ [23]. Robinson et al., suggested that Au nanocrystals act via pathways involving energy metabolism [24]. They reported that nanocrystalline gold (CNM-Au8; 13 nm median diameter) enhanced differentiation of primary OPCs from mouse and rat CNS into mature ODCs with complex cytoplasmic networks [24]. According to their study, levels of NAD+, intracellular ATP, and extracellular lactate were increased, and myelin-synthesis related genes were upregulated in Au-nanocrystal-treated neural cells [24].

Our results showed that almost all cells differentiated on AuNR surfaces, compared to PDL, where only 64% of total cells were differentiated after one week. Among the total population of differentiated and undifferentiated cells, about 41% and 32% of cells were RIP+ ODCs on AuNR and PDL coatings, respectively. Rapid differentiation of NSCs on AuNR surfaces in this case may have been influenced by the interaction between NSCs and AuNRs, as shown by our previous study [6], although the exact mechanism is unknown. Studies have suggested that AuNPs could promote cellular differentiation by activating the mitogen-activated protein kinase (MAPK) pathway [25,26]. Results from the Yang group have shown that AuNPs can be internalized into the cytoplasm of mesenchymal stem cells [25] and osteoblasts [26], activating the MAPK pathway and resulting in their osteogenic differentiation. We do not expect that AuNRs were internalized into NSCs in our study because the AuNRs were attached to a large coverslip. However, the strong interaction of nanoparticle surface topography with the cell membrane could also influence cellular behavior. Surface topography can play essential roles in cell adhesion by regulating cellular transmembrane integrins, which subsequently mediate the secretion of cell-adhesive proteins, such as fibronectin and vitronectin, and formation of focal adhesion [27]. Integrin-activated focal adhesion kinase (FAK) has been shown to play a role in mesenchymal stem cell differentiation [28]. Integrin expression also plays a vital role in ODC differentiation and maturation via Fyn kinase, which activates downward signaling pathways involved in cytoskeletal remodeling during ODC development [17]. However, we did not detect any difference in FAK expression in the differentiating cells on the AuNR surfaces compared to that on PDL after one week in culture. With that said, we could not rule out the possibility that the activity and function of integrin or FAK could have changed, and/or other signaling molecules mediating integrin effects, such as integrin-linked kinase, Akt, Fyn, Rac1, Cdc42, and Rho [17]. It is also possible that other intracellular adaptor proteins, such as talin and vinculin [29], may play more critical roles in the accelerated ODC differentiation.

Besides integrin, the mechanical properties of the extracellular matrix, such as mechanical strain and the stiffness of AuNRs, may have supported more ODC differentiation. Jagielska et al., reported that tensile strain in the range of 10–15% inhibited proliferation of rat OPCs and promoted differentiation of OPCs into ODCs [30]. In another study, Lourenco et al., showed that a substrate stiffness of 6.5 kPa, along with laminin-2/merosin, significantly improved ODC differentiation [31]. In the future, we will characterize and optimize these properties of our AuNR materials to promote more complete differentiation of NSCs toward ODC lineage.

## 5. Conclusions

Our results show that functionalized AuNR substrates are non-toxic to NSCs and promote adhesion and differentiation of NSCs into ODCs and astrocytes. Compared to conventional PDL-coated surfaces, the AuNR surfaces accelerate the differentiation of NSCs in vitro. We did not detect any difference in the expression of integrin and FAK on the AuNR surfaces compared to the PDL surfaces. Further investigations of gene expression of differentiated cells on AuNR substrates and the influence of topography of AuNR surfaces on ODC differentiation and maturation will be performed to delineate the underlying mechanism(s) of NSC differentiation on AuNR surfaces.

## Data Availability

Data are stored at the laboratories of A.S.B. and J.Y.X. and can be obtained upon review of the request.
